# The global health challenge of counterfeit vaccination certificates: The case of yellow fever vaccination among travelers departing from Khartoum International Airport in Sudan

**DOI:** 10.1002/puh2.45

**Published:** 2022-12-07

**Authors:** Razan Osman Abdalla, Salwa Abdulrahman Yousif, Ahmed Tagelsir, Marwa Akola, Alaa Almaleeh, Yasir Ahmed Mohammed Elhadi, Majdi M. Sabahelzain

**Affiliations:** ^1^ Public Health Department School of Health Sciences Ahfad University for Women Omdurman Sudan; ^2^ Clinical Pharmacy Division Aldoha Specialized Hospital Khartoum Sudan; ^3^ Public Health Training and Research Unit Nutrition and Health Center for Training and Research Ahfad University for Women Omdurman Sudan; ^4^ MSc Biotechnology Program School of Pharmacy Ahfad University for Women Omdurman Sudan; ^5^ Public Health Department Sudanese Medical Research Association Khartoum Sudan

**Keywords:** fake vaccination certificates, global health security health policy, International Health Regulations, Sudan

## Abstract

**Background:**

Counterfeit travel vaccination certificates pose a significant threat to public health and compromise disease control measures. We investigated the self‐reported vaccination status, the validity of yellow fever vaccine (YFV) certificates (ICVPs), and knowledge and perception about the disease and its vaccine among Sudanese travelers departing through the Khartoum International Airport (KIA).

**Methods:**

This was a cross‐sectional survey using a non‐probability convenience sampling technique among Sudanese travelers departing through KIA between October and November 2021. We assessed the self‐reported uptake of YFV and subsequently verified the ICVPs among those reported to be vaccinated. We also assessed participants’ knowledge about the disease in addition to their knowledge and perception of YFV.

**Results:**

Four hundred travelers were interviewed. Most participants (88.0%) reported being vaccinated. However, two‐thirds of their ICVPs (63.0%) were counterfeit. More than one‐third of the participants (35.3%) had poor knowledge of YF and YFV. Carrying a valid ICVP was associated with participants' overall good level of knowledge about YFV (*p* = 0.030), knowledge about the nature of infection (*p*‐value = 0.008), disease transmission (*p =* 0.016) and prevention (*p* = 0.028), and countries that require a mandatory YFV proof (*p* = 0.005). Many participants perceived YFV as important (93.3%), safe (86.5%), and effective (82.0%).

**Conclusion:**

The proportion of counterfeit ICVPs was high. Many of the participants had a poor level of knowledge about YF and YFV. Counterfeit ICVPs have grievous implications for YF prevention and control in Sudan and other countries. There is an urgent need to revisit the implementation of the International Health Regulations in Sudan to leverage recent technological advancements in immunization information systems such as electronic certification.

## Graphical abstract

This study investigated self‐reported vaccination status and the validity of the International Certificate of Vaccination or Prophylaxis (ICVPs) among departing Sudanese travelers at Khartoum International Airport in Sudan, 2021. Most participants (88.0%) claimed being vaccinated against Yellow Fever. However, two‐thirds of their ICVPs (63.0%) were counterfeit compared to a standard ICVP issued by health authorities in Sudan.

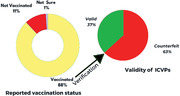

## I**NTRODUCTION**

Yellow Fever (YF) is an acute infectious disease caused by a flavivirus transmitted by the *Aedes* and *Haemogogus* mosquitos [1, 2]. It can be asymptomatic or mild. However, in severe cases, the viral infection causes bleeding, as well as multi‐organ failure of the liver, kidneys, and heart, and might cause death [1, 3, 4]. As there is no specific treatment for YF, the disease is primarily controlled through vaccination of people at risk of infection and vector control [3, 5].

Approximately 200,000 cases of YF are estimated to occur annually, with nearly 90% of these cases identified in Africa, although the risk also exists in tropical and sub‐tropical South America, parts of Central America, and Trinidad in the Caribbean. The mortality rate is considered high, with 30,000 deaths annually [3, 4, 5].

Yellow Fever vaccination (YFV) is well tolerated by most people as it is live attenuated [1, 6]. Since its development in the 1930s, more than 600 million individuals, have been vaccinated worldwide with proven success in protection against yellow fever [5]. The World Health Organization (WHO) recommends several strategies for vaccination to prevent YF, including routine infant immunization, mass vaccination campaigns in countries at risk, and vaccination of travelers going to YF‐endemic areas [1, 6, 7]. For international travelers, the International Health Regulations (IHR) recommend YFV [5, 8]. The International Certificate of Vaccination or Prophylaxis (ICVP) or the Yellow Card is an authenticated paper record that serves as proof of vaccination [4, 8].

Counterfeit travel vaccination certificates can compromise lives and jeopardize disease control to a great extent. This issue has been reported in several countries of Sub‐Saharan Africa; for example, it was stated that approximately 80% of ICVPs in Zimbabwe were counterfeit. In addition, travelers in Uganda can also obtain forged YFV cards. For instance, in October 2016, 50 fake ICVPs were recorded at Entebbe International Airport daily [9]. Tanzania has also acknowledged the existence of syndicates selling falsified ICVPs to arriving tourists [10, 11]. Failure of a traveler to provide the vaccine certificate may result in quarantine for several days, refusal of entry, or vaccination on‐site [7, 8, 12]. Due to the difficulty in validating the ICVP, some countries, such as Nigeria, developed a digital “e‐Yellow Card” that can be verified by scanning a quick response (QR) code to check its validity before traveling outside the country [11]. There is a lack of evidence regarding the validity of ICVPs as well as the scope and magnitude of their fabrication problem in Sudan. This study aimed to investigate self‐reported vaccination status, the validity of ICVPs, knowledge about the disease, and perception of YFV amongst a sample of departing travelers at the largest airport in Sudan.

## METHODS

### Study design and sampling

This study was a cross‐sectional study reported using the Strengthening the Reporting of Observational Studies in Epidemiology (STROBE) statement [13]. The study was conducted among Sudanese travelers from October 15 to November 15, 2021 at the Khartoum International Airport (KIA), the largest airport in Sudan located in the capital city.

The study population included Sudanese travelers departing through the KIA aged 18 years and older and traveling to countries that require a mandatory YFV with an ICVP as proof of being vaccinated. The sample size was calculated based on the average number of travelers per year (25,32,247 in 2021) using EPI‐Info‐7 software based on the following assumptions: 50% expected proportion of counterfeit vaccination certificates and an alpha error of 0.05 and a margin of error of 5%. The minimum required sample size was 384, and a total of 400 participants were interviewed using the non‐probability convenience sampling technique.

### Study variables and data collection

Data were collected using a structured questionnaire administered in the Arabic language. The questionnaires were distributed at the departure gate of KIA while travelers were waiting to board. The questionnaire was self‐administered; nevertheless, two well‐trained interviewers/researchers collected the data from subjects unable to read. The questionnaire included only closed‐ended questions about travelers’ characteristics and destinations. For the Knowledge assessment regarding YF and YFV, we asked 10 questions (Yes/No/Don't know). Perceptions of YFV were measured using 5 points Likert scale (Strongly disagree/Strongly agree).

The self‐reporting of YFV status was first assessed among those who reported being vaccinated, and the validity of the YFV certificates was verified by two well‐trained researchers against the standardized ICVPs approved by the health authority. The criteria used in the visual inspection of ICVP included the documentation of vaccination, date of administration, designation and signature of the clinician (in handwriting), manufacturing and batch number, and the stamp of the administering center.

Fabricated, fake, or manipulated vaccination cards that were not identical to the ICVP issued by the Ministry of Health in Sudan and those that did not satisfy one or more of the above‐mentioned criteria used during visual inspection (certificates with incomplete information) were considered “counterfeit or invalid certificates.” Those that satisfied all the above‐mentioned criteria on visual inspection and those identical to the standardized ICVP issued by the Ministry of Health in Sudan (with correct and complete information) were considered “valid certificates.”

### Data analysis

Data were entered into a Microsoft Excel spreadsheet and then analyzed using the Statistical Package for Social Sciences (SPSS‐v24). For knowledge assessment, each of these 10 items was scored as follows: Yes was assigned a score of 1, while "don't know,“ or No” were assigned a score of 0. The raw total knowledge score was calculated by simply summing each item. The total raw score ranged from 0 to 10. The knowledge scores were then grouped into three categories 0–3 (Poor), 4–6 (Average), and 7–10 (Good). Descriptive statistics and chi‐square tests were performed. Those with *p* < 0.05 were considered significant.

### Ethical consideration

This study was carried out following the ethical standards outlined in the Helsinki Declaration and its subsequent amendments or comparable ethical standards [14]. The Institutional Review Board approval was obtained from Ahfad University for Women's, Omdurman, Sudan. The KIA administration office approved the permission to enter the airport of KIA. Confidentiality and anonymity were assured by coding questionaries and masking the personal details from the photocopies of participants’ ICVPs before the assessment and authentication. Informed written consent was obtained from all participants before the commencement of the study and after the explanation of the purpose and benefits of the study, and clearly stating the confidentiality of the results and voluntary participation in the study.

## RESULTS

### Travelers’ profile and destination

Out of 400 travelers, more than half (55.5%) were males, and nearly half of them (48.4%) had a university education, 22.0% were students, and 21.8% were specialized professionals (doctors, lawyers, and engineers). Most of the travelers (80.8%) stated that this was not their first time traveling, and the majority of the participants (80.0%) were traveling to Egypt (Table 1).

**TABLE 1:** Characteristics of departing Sudanese travelers surveyed for self‐reported YFV status and the validity of ICVPs at Khartoum International Airport, Sudan, 2021 (*n* = 400)

Travelers' profile and destination	*n*	(%)
Sex		
Female	192	(48.6)
Male	203	(51.4)
Education level		
Cannot read and write	11	(2.8)
Primary school	39	(9.9)
Secondary school	154	(39.0)
University degree	191	(48.4)
Employment		
Housewife	49	(12.4)
Student	87	(22.0)
Worker	26	(6.6)
Officer	78	(19.7)
Specialized professions (Doctor, Lawyer, Engineer)	86	(21.8)
Self‐employed (booth, restaurant, tea place, company)	69	(17.5)
First‐time travelers	76	(19.2)
Destination		
Egypt	316	(80.0)
Kenya	30	(7.6)
Uganda	14	(3.5)
Djibouti	10	(2.5)
Nigeria	10	(2.5)
Tanzania	7	(1.8)
Rwanda	8	(2.0)

### Vaccination status and verification of ICVPs

**FIGURE 1:**
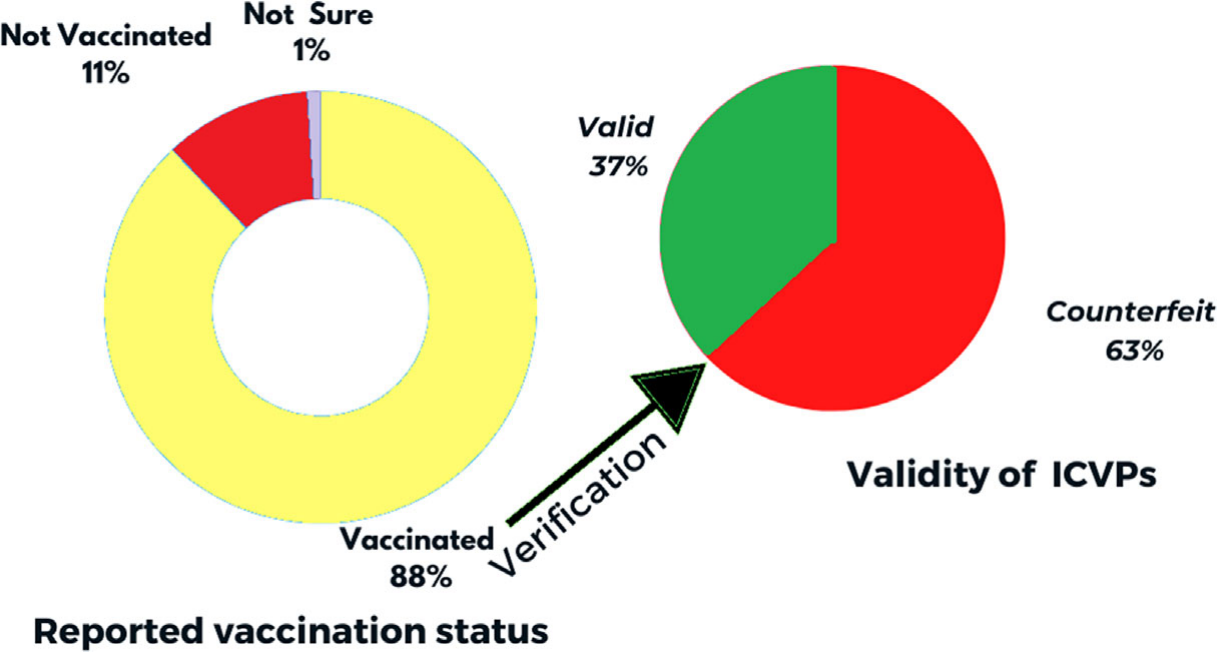
Self‐reported YFV status and subsequent verification international certificate of vaccination or prophylaxis (ICVPs) among departing Sudanese travelers at Khartoum International Airport, Sudan, 2021.

As demonstrated in Figure 1, most of the respondents (88.0%) reported having received vaccination against YF, of which 63.0% of their ICVPs were fake upon verification and comparison with the standard ICVPs approved by authority. While 11.0% reported to be not vaccinated against YF and 1.0% were unsure.

### Knowledge about YF and YFV and their association with the validity of ICVP

The results of the knowledge assessment shown in Table 2 indicate that about half of the participants (47.7%) had good knowledge regarding YF and YFV, while more than one‐third of the participants (35.3%) had poor knowledge, and 17.0% had average knowledge scores. In addition, among participants having fake ICVPs, more than two‐thirds (68.8%) had a total poor knowledge score. Moreover, there was a significant association between the validity of ICVPs and participants’ total knowledge scores (*p* = 0.030), knowledge about the nature of YF infection (*p* = 0.008), disease transmission (*p* = 0.016), disease prevention through vaccination (*p* = 0.028), and countries that require a mandatory YFV (*p* = 0.005).

**TABLE 2:** Knowledge of YF and YFV among travelers departing through the Khartoum International Airport, Sudan 2021 (*n* = 400).

**Variables**		**Validity of ICVPs**				***p*‐value**
		**Valid**		**Counterfeit**		
		*n*	(%)	*n*	(%)	
Total scores of participants' knowledge	Poor knowledge	44	(31.2)	97	(68.8)	**0.030**
	Average knowledge	26	(38.2)	42	(61.8)	
	Good knowledge	87	(45.5)	104	(54.5)	
Yellow Fever is a viral disease	Yes	111	(44.2)	140	(55.8)	**0.008**
	No	46	(30.9)	103	(69.1)	
Yellow Fever transmitted by mosquitoes	Yes	57	(48.3)	61	(51.7)	**0.016**
	No	100	(35.5)	182	(64.5)	
Bleeding dangerous symptom of Yellow Fever	Yes	68	(44.4)	85	(55.6)	0.094
	No	89	(36.0)	158	(64.0)	
Sudan is one of the Yellow Fever endemic countries	Yes	82	(42.1)	113	(57.9)	0.263
	No	75	(36.6)	130	(63.4)	
Yellow Fever can be prevented through vaccination	Yes	131	(42.1)	180	(57.9)	**0.028**
	No	26	(29.2)	63	(70.8)	
The Yellow Fever vaccine contains a weak live virus	Yes	90	(42.3)	123	(57.7)	0.322
	No	67	(36.0)	119	(64.0)	
One dose of the Yellow Fever vaccine can provide a lifetime protection	Yes	57	(35.8)	102	(64.2)	0.329
	No	99	(41.4)	140	(58.6)	
Yellow Fever vaccine should not be given to people who are allergic to eggs	Yes	35	(36.1)	62	(63.9)	0.463
	No	122	(40.3)	181	(59.7)	
In some countries, Yellow Fever vaccination is a mandatory requirement	Yes	148	(41.7)	207	(58.3)	**0.005**
	No	9	(20.0)	36	(80.0)	
Yellow Fever vaccination cards are sold at many travel agencies without receiving the vaccine	Yes	92	(43.4)	120	(56.6)	0.071
	No	65	(34.6)	123	(65.4)	

Bold values are statistically significant. Abbreviation: ICVP, International Certificate of Vaccination or Prophylaxis.

### Participants’ perception of YFV

The majority of participants agreed/strongly agreed that vaccination against the disease is important (93.3%), safe (86.5%), and effective (82.0%). However, the percentage of participants who strongly disagreed with the importance and safety of YFV was relatively higher among those having counterfeit yellow cards compared to those with valid ICVPs (Table 3).

**TABLE 3:** Perception of YFV among travelers departing through the Khartoum International Airport, Sudan 2021 (N = 400)

**Variables**	**Responses**	**Total**	**Validity of ICVPs**			
			Valid		Counterfeit	
			*n*	(%)	*n*	(%)
I think vaccination against Yellow Fever is important	Strongly disagree	6	0	(0.0)	6	(100.0)
	Disagree	2	1	(50.0)	1	(50.0)
	Not sure	19	11	(57.9)	8	(42.1)
	Agree	103	34	(33.0)	69	(67.0)
	Strongly agree	270	111	(41.1)	159	(58.9)
I think vaccination against Yellow Fever is safe	Strongly disagree	5	0	(0.0)	5	(100.0)
	Disagree	3	1	(33.3)	2	(66.7)
	Not sure	46	22	(47.8)	24	(52.2)
	Agree	118	40	(33.9)	78	(66.1)
	Strongly agree	228	94	(41.2)	134	(58.8)
I think vaccination against Yellow Fever is effective	Strongly disagree	5	1	(20.0)	4	(80.0)
	Disagree	4	0	(0.0)	4	(100.0)
	Not sure	63	22	(34.9)	41	(65.1)
	Agree	123	45	(36.6)	78	(63.4)
	Strongly agree	205	89	(43.4)	116	(56.6)

Abbreviation: ICVP, International Certificate of Vaccination or Prophylaxis.

## DISCUSSION

On May 23, 2005, the model ICVP replaced the International Certificate of Vaccination or Revaccination against YF [15]. YFV is the most common vaccine required for international travel. Many countries require the YFV for all visitors or only those coming from countries with a high risk of disease transmission [16]. Depending on the country, newborns are usually exempt until they are 9 months or 1 year old. Before 2016 YFV was valid for 10 years; however, now it is considered valid for the entire life, and thus there are no changes required for those who received their ICVP before 2016 [17].

Counterfeit travel vaccination certificates represent a challenging public health threat that impedes the massive efforts to control and prevent communicable diseases worldwide. Among Sudanese travelers, most participants (88%) reported being vaccinated, however, nearly two‐thirds of their ICVPs (63.0%) were counterfeit or invalid certificates. Sudan has a black market for vaccination cards, with the certificate costing 8 USD, even though the YFV costs about 25 USD [12]. To eliminate the risk of fake vaccination cards, the WHO, in collaboration with national stakeholders in Sudan, should revisit the implementation of IHR and enforce effective governmental laws and policies. This is a global health issue as counterfeit vaccine certificates were reported in Zimbabwe and Nigeria [10, 13]. Disease risk behaviors such as fabricating ICVPs are considered a deliberate violation of the IHR as they can lead to a dramatic resurgence of vaccine‐preventable diseases and put lives at risk [9]. Moreover, the presence of a considerable proportion of participants in the current study who traveled without vaccination (11.0%) is a significant public health threat especially since they are traveling from one of the most endemic areas heading different countries around the globe. Similarly, a prospective study conducted at Travel Health Clinic in Spain showed that more than a third of participants traveled without getting YFV [18].

According to WHO data, the YFV was successfully administered to approximately 75% of the Sudanese population aged 9 months to 60 years in 2019, and it was included in the routine schedule of immunization of infants when they turn 9 months [19]. As the WHO considers an 80% vaccine coverage effective in preventing YF outbreaks, it is necessary to assess the YFV rate in Sudan, particularly among travelers to overseas countries. In addition, these findings raise several concerns about the global acceptance of the paper‐form ICVPs as proof of travelers’ vaccination status and re‐iterate the urgent need to include more recent technological advancements in immunization information systems such as electronic certification [20]. However, the experience of Nigeria shows that electronic certification alone is not enough to curb this issue. Even after the implementation of an e‐registry system to fight this issue, people can still obtain a government‐issued certificate without vaccination [10]. This highlights the urgent need for multifaceted policy and health behavior interventions to tackle this challenge. An in‐depth understanding of the reasons for avoiding YF vaccination is critical as implementing a policy intervention without interventions to mitigate the root causes and underpinnings of the problem is unlikely to be effective.

Concerning factors associated with the validity of respondents’ ICVPs, the current study revealed a significant association with the participant's overall good level of knowledge about YF and YFV, as the highest proportion of counterfeit ICVPs were detected among participants with poor knowledge about YF and YFV. Further, it was significantly associated with knowledge about the nature of YF infection, disease transmission, disease prevention through vaccination, and countries that require a mandatory YFV. Knowledge about the disease is essential to disease control. Indeed, the control and prevention of infectious diseases depend on a thorough understanding of disease severity, preventive measures, and factors influencing transmission [21]. This result should prompt stakeholders to implement targeted health education interventions to increase travelers' disease awareness and promote YFV in Sudan.

While most participants in the current study perceived YFV as important, safe, and effective, a substantial proportion preferred the fabricated ICVPs rather than the vaccination and obtaining a valid ICVP. This could be explained by the perceived accessibility and affordability of travel certificates. Fake ICVP is relatively easier to obtain as valid ICVP is only issued at Sudanese Medical Commission centers. Perceived unavailability of vaccination services and certificates especially in rural areas in Sudan might have encouraged participants to go for counterfeit ICVPs. Also, the unaffordable price of ICVPs may be another contributing factor. Overall, these results should draw the attention of policy leaders in Sudan to expand the availability of YFV certification centers, especially in rural and peripheral areas of the country.

### Limitations

This study is susceptible to misclassification bias because the validity of ICVP was assessed visually. Furthermore, our study population consisted of international travelers leaving Sudan, so generalizing the findings to other populations may be limited.

## CONCLUSIONS

A substantial proportion of departing Sudanese travelers had counterfeit ICVPs, with many of the participants demonstrating a poor level of knowledge about YF and YFV. The knowledge about the disease and vaccination was associated with the possession of valid ICVPS. Counterfeit ICVPs have grievous implications for yellow fever prevention and control in Sudan and other countries. There is an urgent need to revisit the implementation of the International Health Regulations in Sudan to leverage recent technological advancements in immunization information systems such as electronic certification.

## AUTHOR CONTRIBUTIONS

Razan Abdalla and Majdi Sabahelzain developed the concept of this study; Majdi Sabahelzain and Yasir Elhadi wrote the first draft. Razan Abdalla, Salwa Yousif, Ahmed Tagelsir, Marwa Akola, and Alaa Almaleeh collected the data and prepared the manuscript. All authors contributed to writing‐review and editing and approved the final version for submission.

## CONFLICT OF INTEREST

Elhadi Y.A.M is a Youth Editorial Board member of Public Health Challenges and co‐author of this article. He was excluded from editorial decision‐making related to the acceptance of this article for publication in the journal.

## ETHICS STATEMENT

The Institutional Review Board approval was obtained from Ahfad University for Women. The permission for entering the airport was approved by the managerial office of KIA.

## Data Availability

The data that support the findings of this study are available from the corresponding author upon reasonable request.
